# Mobile Health for Perinatal Depression and Anxiety: Scoping Review

**DOI:** 10.2196/17011

**Published:** 2020-04-13

**Authors:** Neesha Hussain-Shamsy, Amika Shah, Simone N Vigod, Juveria Zaheer, Emily Seto

**Affiliations:** 1 Institute of Health Policy, Management, and Evaluation Dalla Lana School of Public Health University of Toronto Toronto, ON Canada; 2 Centre for eHealth Global Innovation Techna Institute University Health Network Toronto, ON Canada; 3 Women's College Hospital and Women's College Research Institute Toronto, ON Canada; 4 Department of Psychiatry University of Toronto Toronto, ON Canada; 5 Centre for Addiction and Mental Health Toronto, ON Canada

**Keywords:** mental health, depression, anxiety, pregnancy, postpartum, smartphone, mobile phone, text message, mHealth

## Abstract

**Background:**

The perinatal period is a vulnerable time during which depression and anxiety commonly occur. If left untreated or undertreated, there may be significant adverse effects; therefore, access to rapid, effective treatment is essential. Treatments for mild-to-moderate symptoms according to a stepped-care approach involve psychoeducation, peer support, and psychological therapy, all of which have been shown to be efficaciously delivered through digital means. Women experience significant barriers to care because of system- and individual-level factors, such as cost, accessibility, and availability of childcare. The use of mobile phones is widespread in this population, and the delivery of mental health services via mobile phones has been suggested as a means of reducing barriers.

**Objective:**

This study aimed to understand the extent, range, and nature of mobile health (mHealth) tools for prevention, screening, and treatment of perinatal depression and anxiety in order to identify gaps and inform opportunities for future work.

**Methods:**

Using a scoping review framework, 4 databases were searched for terms related to mobile phones, perinatal period, and either depression or anxiety. A total of 477 unique records were retrieved, 81 of which were reviewed by full text. Peer-reviewed publications were included if they described the population as women pregnant or up to 1 year postpartum and a tool explicitly delivered via a mobile phone for preventing, screening, or treating depression or anxiety. Studies published in 2007 or earlier, not in English, or as case reports were excluded.

**Results:**

A total of 26 publications describing 22 unique studies were included (77% published after 2017). mHealth apps were slightly more common than texting-based interventions (12/22, 54% vs 10/22, 45%). Most tools were for either depression (12/22, 54%) or anxiety and depression (9/22, 41%); 1 tool was for anxiety only (1/22, 4%). Interventions starting in pregnancy and continuing into the postpartum period were rare (2/22, 9%). Tools were for prevention (10/22, 45%), screening (6/22, 27%), and treatment (6/22, 27%). Interventions delivered included psychoeducation (16/22, 73%), peer support (4/22, 18%), and psychological therapy (4/22, 18%). Cost was measured in 14% (3/22) studies.

**Conclusions:**

Future work in this growing area should incorporate active psychological treatment, address continuity of care across the perinatal period, and consider clinical sustainability to realize the potential of mHealth.

## Introduction

### Background

The perinatal period, often defined as the period between conception and up to 1 year after giving birth, is a vulnerable time during which depression and anxiety commonly occur. Depression and anxiety during this period of time affect 14.5% and 13% of women, respectively, and these are comorbid among 8.1% of new mothers [1-3]. If left untreated or undertreated, there are serious, adverse short- and long-term personal, social, and economic consequences for the mother, child, family unit, and society [4,5]. Access to prevention strategies, screening tools, and rapid and effective treatment is essential.

During the perinatal period, effective treatments may include pharmacological (ie, antidepressant) or psychological treatment, depending on the severity of illness and individual treatment preferences [6]. A stepped-care approach, reflecting high-intensity treatment as illness severity progresses, is recommended [7,8]. This involves a first step involving psychosocial interventions (eg, psychoeducation and peer support), followed by a second step to psychological therapies for illness of mild-to-moderate severity, as well as pharmacological and other biological treatments (ie, antidepressants) as a third step for more severe and/or lasting illness. The risks of antidepressant use in pregnancy are low (selective serotonin reuptake inhibitors are first-line drugs in pregnancy, but serotonin-norepinephrine reuptake inhibitors, bupropion, and mirtazapine can also be used in some circumstances) and must be weighed against the risks and benefits of all other treatment options (eg, no treatment or psychosocial, psychological, or pharmacological treatment alone or in combination) [9,10]. System-level barriers to accessing evidence-based care in this population include long wait lists for specialty psychiatric care and cost and availability of specialists [11]. This population also has unique practical barriers to accessing care, such as the cost and availability of childcare, transportation, and difficulty in scheduling appointments around an infant’s often unpredictable needs [12,13].

Electronic health (eHealth) is a broad term used to describe the use of information and communication technologies for health, under which falls mobile health (mHealth) [14]. mHealth specifically relates to the use of mobile computing and communication technologies for health purposes, and it may include apps and text messaging–based (SMS) interventions as well as wearable devices [15]. In some cases, these can be developed and implemented at low cost [16]. These have the potential to help address barriers to accessing care, partly as they offer a greater level of portability and convenience for this population in a way that internet-based interventions designed for access on stationary devices (eg, desktop and laptop computers) cannot [17-19]. In high-income countries, mobile phone ownership is greater than 90%; smartphone (ie, a mobile phone that can access the internet and mobile applications) ownership is as high as 76%, with the highest rates of use among those under 35 years (in other words, who are also in their prime reproductive years) [20]. It is increasingly common to exclusively access the internet via a mobile device [21]. Rates of use and acceptance of mHealth interventions may differ based on a variety of individual factors including age, location (eg, developing country or not), and type of health outcome [22,23].

mHealth tools have the potential to be scalable, cost-effective, and to simultaneously benefit individual patients and the health care system [18,23]. To support mental health in the general population, mHealth tools are used for appointment and medication reminders, information and education, providing support, and self-monitoring. These have also been found to be effective at reducing symptoms when delivering psychotherapy and other behavioral interventions, although they have historically been used outside of the health care system [17,24-27]. Psychological treatments, such as cognitive behavioral therapy (CBT) and mindfulness, behavioral activation, and interpersonal therapy, are evidence-based treatments that can be efficaciously delivered in person and in digital formats, with emerging evidence to support their delivery via mHealth tools [28-30]. mHealth tools could tailor these interventions for perinatal populations to address the gaps in accessing mental health care. Publicly available apps to address postpartum depression were assessed to be of extremely low information quality [27]. The ubiquity of mobile phones, coupled with their potential to deliver effective psychosocial and psychological interventions, makes them a promising avenue through which to address the barriers to care in the delivery of mental health services among women with perinatal depression and anxiety.

### Objective

Given the high rate of comorbidities and their similar treatment options, a comprehensive understanding of the current state of mHealth tools to support women with either perinatal depression or anxiety would be helpful to inform potential future work, but this understanding does not yet exist. The objective of this scoping review was to summarize the extent, range, and nature of academic literature about the use of mHealth tools for the prevention, screening, and treatment of perinatal depression and anxiety. Understanding the current state of the art through the results of this review will help identify the gaps in knowledge and practice, thereby informing opportunities for future work.

## Methods

### Study Design

A scoping review framework, defined by Arksey and O’Malley [31] and further developed by Levac et al [32], was used for this study. A scoping review was the most appropriate manner of assessing the breadth of the literature and identifying gaps and opportunities for further work in this field [33].

### Databases and Search Terms

Four databases (Medical Literature Analysis and Retrieval System Online, EMBASE, PsycINFO, and Cumulative Index to Nursing and Allied Health Literature Plus) were searched for terms related to the following: (1) mobile phones, (2) the perinatal period (eg, pregnancy, postpartum, antenatal, and maternal), and (3) depression or anxiety (alone or in combination). Each database was individually searched by title, abstract, keyword, and subject heading for relevant search terms. A librarian specializing in systematic and scoping review search strategies reviewed the search strings. Rayyan (Qatar Computing Research Institute, Hamad bin Khalifa University), a Web-based platform designed for data management for systematic reviews, was used to manage citations and filter results [34].

### Inclusion and Exclusion Criteria

Studies were included if they met the following criteria: (1) defined the primary study population as women who were pregnant or had given birth in the past 12 months; (2) defined perinatal depression or anxiety as the outcome of interest for the intervention; (3) described a study (through a protocol or presentation of study results) related to a tool intentionally and primarily delivered through a mobile phone (including, but not limited to apps, SMS text messaging–based interventions) for the prevention, screening, or treatment of perinatal depression and/or anxiety; (4) were published in a peer-reviewed journal; and (5) intended to recruit (for study protocols) or reported a study sample of >1. Studies were excluded if they met the following criteria: (1) published in 2007 or earlier and (2) published in a language other than English (because of resource limitations in finding translations). The time restriction of articles published after 2007 or earlier reflects the upsurge in publications on mHealth care after this date, as a result of the introduction of the Apple iPhone [15]. Both quantitative and qualitative studies were eligible for inclusion provided all other criteria were met.

### Article Selection and Abstraction

Hand searches were conducted by one reviewer (NH-S) who reviewed the reference lists of relevant scoping reviews, systematic reviews, and meta-analyses, as well as reference lists of all publications included in the study for any additional relevant articles.

Two stages of iterative screening for article selection were conducted [32]. In the first stage of screening after the removal of duplicate articles, two reviewers (NH-S and AS) independently assessed articles by title and abstract for potential inclusion in the study. Any articles that were clearly not relevant to this scoping review were excluded. In the second stage of screening, the same reviewers independently applied inclusion and exclusion criteria to all articles (including those found through hand searching) based on a full-text reading of each article. Both reviewers independently conducted data abstraction using an iterative process. Discrepancies in decisions at each stage were resolved through discussion and consensus. The initial search was conducted in February 2019, for a period spanning from 2007 to the search date. The search was updated to ensure capturing any new publications made between February 2019 and July 2019. These articles were selected using the same two-stage selection process described above.

## Results

### Selected Articles

**Figure 1:**
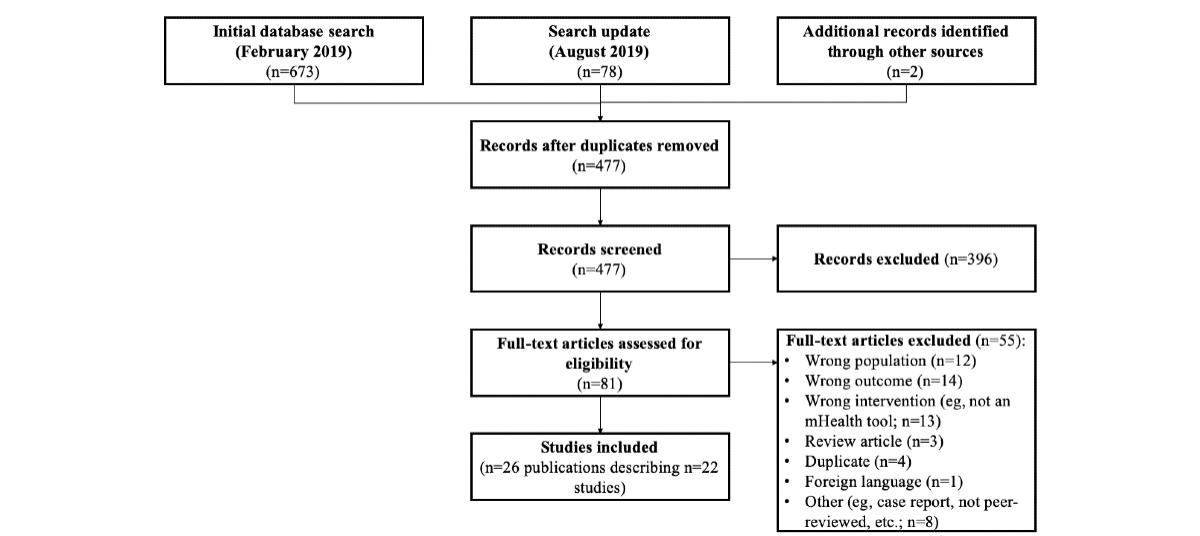
Preferred Reporting Items for Systematic Review and Meta-Analyses flowchart of study selection.

The initial search of four databases yielded 673 results. An additional 78 records were retrieved in the search update (Figure 1). Hand searching identified 2 additional records. After the removal of duplicate publications, 477 publications (427 from initial search, 48 from the search update, and 2 from hand searching) were reviewed. On the basis of title and abstract, 396 articles were excluded, including 13 systematic reviews whose reference lists were searched. Of the 81 publications reviewed by full text, 55 publications were excluded (wrong population, n=12; intervention targeting an outcome other than depression or anxiety, n=14; nonmobile phone–based intervention, n=13; not in English, n=1; review article, n=3; not peer reviewed, n=6; and duplicate, n=4). Hand searching of the reference lists of the reviews identified in full-text screening identified no additional relevant publications.

Overall, 26 publications (22 papers and 4 conference abstracts) describing 22 unique studies were included in this review. Of these publications, 5 were study protocols for which no results were available. Studies varied by the type of technology used (eg, SMS or app), purpose of the tool (eg, either prevention, screening, or treatment), illness (eg, depression or anxiety), and time point (eg, pregnancy and/or postpartum).

### Population

Interventions were limited to be delivered either during pregnancy (7/22, 32%) [35-43] or in the postpartum period (10/22, 45%) [44-54]. One intervention (4%) was designed to be used during pregnancy or postpartum [55,56], and 2 interventions (9%) were designed to begin during pregnancy and continue into the postpartum period [57,58]. For an additional 2 studies (9%), the time period was unclear [59,60]. A total of 7 studies (32%) targeted their interventions to marginalized populations with potentially higher needs as compared with the general population. In all, 2 studies did so through direct approaches and targeted recruitment to low-income women and adolescent mothers [36,57]. A total of 4 studies did so indirectly by recruiting from centers that predominantly service marginalized populations [39,42,43,48,53,54]. One study indirectly included high-risk populations by specifically noting that women who had given birth to multiples or with an infant admitted to a neonatal intensive care unit (NICU) would not be excluded, as those mothers are at high risk for depression and anxiety [47]. Mothers with high-risk pregnancies or infants admitted into the NICU were often specifically excluded from other studies.

### Intervention Purpose and Outcome

The purposes of the interventions in the included studies were the following: (1) prevention (10/22, 45%; Multimedia Appendix 1) [35-39,47,49,52,57,59], (2) screening (6/22, 27%; Multimedia Appendix 2) [40-44,50,55,58], and (3) treatment of perinatal mental illness (6/22, 27%; Multimedia Appendix 2) [45,46,48,51,53,60]. The majority of interventions focused on depression only (12/22, 54%) [36,37,40,41,44,46-48,50,51,53,54,58,60]; 1 study (1/22, 4%) focused only on anxiety [35], and 9 studies (9/22, 41%) focused on both illnesses [38,39,42,43,45,49,52,55,59]. Of the 10 studies in which outcomes were reported, 7 studies (70%) reported a positive impact on mental health [35,36,43,45,46,48,57]. Only 4 studies (4/22, 18%) measured long-term outcomes beyond a postintervention survey.

### Intervention Strategy

Overall, there was a large variation in the types of strategies used by each mHealth tool to achieve its intended purpose, including psychoeducation (16/22, 73%), symptom monitoring (7/22, 32%), and/or peer support (4/22, 18%). A total of 4 studies (18%) used an active psychological therapy (CBT [2/22, 9%], mindfulness-based therapy [1/22, 4%], and attention bias modification training [1/22, 4%]). In all, studies used other strategies, including tracking movement (2/22, 9%) and encouraging exercise (1/22, 4%). Most studies involved a tool that incorporated multiple strategies (11/22, 50%); most frequently, it was psychoeducation in combination with another strategy (9/22, 41%). One preventative study delivered psychoeducation via SMS using text messages informed by CBT principles, but it was specifically noted that it did not deliver a complete CBT intervention; therefore, this study was counted as a psychoeducational intervention only [55,56].

A total of 6 studies (27%) included a component to help facilitate communication with a health care provider [42,43,50,53,54,58,60]. In all, 2 of these studies used passive communication, where the onus lay on the mother to choose to respond to an SMS text message to receive a phone call from a nurse [47,53,54]. A total of 4 studies used more directive communication with the health care provider, where an automatic alert to a health care provider was sent if symptoms were self-reported via the symptom-monitoring questionnaires delivered by app or SMS and were beyond an acceptable threshold [41,50,58,60].

### Study Phase and Research Methodology

Publications described the various phases in the life cycle of such tools from design and development (5/22, 23%), feasibility and acceptability (16/22, 73%), to efficacy and effectiveness (10/22, 45%). Multiple studies had information (through study protocol or results) related to multiple phases of the intervention life cycle. No publications described implementation, scalability, and sustainability efforts. There are an increasing number of efficacy/effectiveness studies over time (Table 1). Most studies (13/22, 59%) solely described quantitative work, whereas only 1 study (1/22, 4%) described research that was solely qualitative in nature. A total of 7 studies (7/22, 32%) described the use of both quantitative and qualitative methods; 1 additional study (1/22, 4%) was explicitly described as being mixed method. Studies were typically designed as randomized controlled trials (RCTs; 9/22, 41%), quasi-experimental (6/22, 27%), or cohort studies (3/22, 13%). Overall, 4 studies (4/22, 18%) incorporated multiple study designs: a cross-sectional survey followed by a quasi-experimental design, a cohort study followed by the development of a machine learning–based algorithm, a longitudinal cohort study culminating in an RCT, and a study described as a qualitative design approach to the development of the tool and intervention.

In all, 3 studies (3/22, 13%) included some measures related to cost, although none provided any data to allow for a comparison between the mobile tool and standard care.

**Table 1:** Study phase over time, based on year of publication (N=22 studies).

Year^a^	Design and development, n (%)	Feasibility and acceptability, n (%)	Effectiveness and efficacy, n (%)
2008	N/A^b^	1 (4)	N/A
2009	N/A	N/A	N/A
2010	N/A	N/A	N/A
2011	N/A	N/A	N/A
2012	N/A	N/A	N/A
2013	1 (4)	1 (4)	1 (4)
2014	1 (4)	2 (9)	N/A
2015	1 (4)	1 (4)	N/A
2016	N/A	N/A	N/A
2017	N/A	2 (9)	1 (4)
2018	2 (9)	5 (23)	4 (18)
2019	N/A	4 (18)	4 (18)

^a^Note that multiple study phases are reported for some studies.

^b^Not applicable.

### Setting

Of the 22 studies, 1 study (4%) was conducted in a lower-middle income economy (Kenya), 3 studies (13%) were conducted in upper-middle income economies (China, Iran, and Thailand), and the remaining studies (18/22, 81%) were conducted in high-income economies (Australia, n=5; Canada, n=2; Spain, n=1; the United Kingdom, n=1; and the United States, n=9), as per their World Bank income group classification [61]. Studies recruited women from a combination of hospitals (13/22, 59%), community-based settings (7/22, 32%), and/or through other means, such as social media or posters (4/22, 18%). One study (1/22, 4%) did not report this information. A total of 8 studies (8/22, 36%) specifically noted that recruitment occurred in an academic hospital or clinic.

### Technology

There were more studies using apps (12/22, 54%) compared with SMS-based tools (10/22, 45%) over time, and there was an increase in the number of studies published after 2017 (17/22, 77%). Half of the apps (6/12, 50%) were developed for both iOS and Android devices or as a Web-app, and the other half of the app-based studies either built tools for a single platform (iOS or Android) or did not report this information. A total of 4 studies (4/22, 18%) were explicit in their use of a user-centered design process.

## Discussion

### Principal Findings

This scoping review found 22 studies related to the prevention, early detection or screening, or treatment of either depression or anxiety in the perinatal period using mobile phones. Interventions most frequently involved psychoeducation and focused on the prevention of illness. Results highlight that research into the use of mHealth tools to support perinatal mental health care is growing and that there are key areas of focus, as described below, for researchers to explore in order for the field to progress.

Results suggest that the potential for mHealth tools to improve access to stepped mental health care for women with either perinatal depression or anxiety is beginning to be realized. The expanded use of mHealth to facilitate delivery of mental health care is most appropriate for women experiencing mild-to-moderate symptoms of depression and anxiety in the perinatal period, which can be addressed using psychosocial and psychological treatments according to a stepped-care approach; the needs of the 11% of women with moderate-to-severe symptoms requiring more intensive clinical management fall beyond the scope of what mobile tools can provide on their own [62,63]. Interventions in this review, which used strategies such as psychoeducation and peer support, are appropriate for prevention and to treat the 53% of women with symptoms that are of mild severity [63]. Many women are already using publicly available apps to access informational support, despite evidence to suggest that the information provided in these apps is incomplete [40,64-66]. The development of mHealth tools in a manner that provides accurate, reliable information and the generation of evidence, as outlined in this review, to show they have the potential to efficaciously prevent or reduce mild symptoms of perinatal depression and/or anxiety is an important positive step.

A major gap highlighted by this review is that only a few tools engaged in the delivery of active psychological therapies (eg, CBT and mindfulness), despite evidence to support the efficacious Web-based delivery of these therapies for general mental health conditions, as well as the emerging evidence to support their delivery via mobile apps [29,67]. These types of strategies and interventions would be beneficial for the 36% of women with postpartum depression who experience symptoms of mild-to-moderate severity [63]. Internet-delivered psychotherapies have lower dropout rates, lower cost, and broader reach than in-person treatment, outcomes that in theory should be maintained or enhanced by mobile delivery, given that it would also address additional practical barriers to care in this population [68]. For example, the use of an artificial intelligence chatbot to deliver a CBT intervention by SMS, included in this review, addresses many challenges in the delivery of perinatal mental health care by being accessible 24×7 to an unlimited number of patients, avoiding scheduling and transportation issues for users and at the same time having health care providers available to address issues beyond the scope of the chatbot’s capacity (eg, talk of self-harm) [60]. In a separate study, when given the option of accessing a peer support group for postpartum women on the Web or through a mobile app, 93.7% of the users opted to use the mobile app, and usage data demonstrated that the majority of app use was between 6 pm and 8 am—a time of the day during which health care providers are not typically available [45]. Modern technological capacity provides the opportunity to develop and use mHealth tools that can address needs in a patient-centered manner, and multiple studies highlighted the interest among both health care professionals and women in the perinatal period to be able to deliver and access mental health support in this manner [69,70].

### Challenges With Design, Evaluation, and Sustainability

Publications included in this review primarily described the design and development of an mHealth tool or assessed feasibility. The lack of scale and implementation studies suggests they have yet to be operationalized and scaled for widespread use, particularly in a scientifically rigorous manner. There may be several reasons for this. First, this is a growing area of research, noted in that the majority of studies were published after 2017 and in the increasing number of efficacy and effectiveness studies over time. It is possible that these efforts are underway but have not yet been described in the literature. Second, assessing efficacy and effectiveness are expensive and time-consuming endeavors, given that RCTs have long been considered the gold standard for determining the efficacy of clinical interventions. However, they are rigid and lengthy to conduct, creating the risk that the tool could be obsolete before scale and implementation can begin [71]. There are calls for the evaluation of mHealth tools, in general, and in psychiatry, in particular, to move toward a model of *naturalistic* evaluation through the use of embedded continuous data collection, in-app user surveys (ie, star rating systems for modules immediately after use), and qualitative interviews, thus enabling an evaluation that is more translational in nature [72,73]. Consideration should also be given to focusing on testing the principles of the intervention versus the technology that is used to allow tools to adapt, while remaining adherent to the treatment embedded within them [74]. This could facilitate rapid scale and implementation of mHealth tools.

The development of any tool with the potential to enhance clinical practice and patient experience should ideally incorporate factors that are likely to contribute to success and sustainability. For eHealth tools, user-level factors related to success are engagement with (eg, personal agency, motivation, and values) and the quality of the intervention itself [75]. At the system level, identified factors for success include those related to improvements in the quality of health care, including facilitating patient-provider communication and supporting patient-centered care, whereas factors related to failure are often relevant to cost, particularly the connection (or lack thereof) between quality of care provided by such tools and their system-wide cost savings [76]. Elements of patient-provider communication were used in less than one-third of the studies in this review, and only one study had a bidirectional chat feature, allowing for ongoing two-way communication with a provider [58]. In the same way that guided internet-based therapies are more effective and have higher adherence than self-directed internet-based interventions, attention should be paid to the best ways of integrating the expertise and capabilities of clinical providers into mHealth tools [77]. Publications included in this review rarely incorporated information on the cost or cost-effectiveness of the mobile tool in comparison with standard of care. Given that both communication and cost have been noted as key factors that influence sustainability, future work should incorporate these elements into the design of the tool and its evaluation.

### Continuity of and Access to Care

Tools in this review address the need for mental health support during either pregnancy or the postpartum period, but these did not offer meaningful opportunities to provide continuity of care across the perinatal period, during which symptoms of depression or anxiety may ebb and flow [78]. Interventions housed in an obstetrics clinic may only be accessible to women currently accessing services, which typically end at 6 weeks postpartum, before the highest-risk period for the onset of symptoms of postpartum depression. Interventions based in clinics that specialize in the treatment of perinatal mental health concerns might be available to women throughout the perinatal period, but these are then limited to those who can be referred to and access these limited services. Most studies included in this review were based in urban, academic settings, which may have led to the passive exclusion of women receiving maternal care in the community (eg, from a midwife) or in rural settings, who may not otherwise have access to specialty perinatal mental health care that is more frequently available in academic centers. Any conclusions made about these tools are therefore potentially limited in their generalizability to urban, academic contexts, which may not reflect the perinatal population in general. Delivery of mental health care services via mobile tools has the potential to remove access barriers for hard-to-reach populations, particularly those outside of urban centers. Future work should address these possibilities by engaging in the recruitment of women with limited access to treatment, in-person or otherwise.

mHealth tools have the potential to address gaps in health care service delivery in low- and high-income economies alike; however, most studies included in this review were conducted in high-income countries. Further investigation is required to understand the nature of mHealth tools being developed and used in lower-middle income economies to address the mental health needs of women in the perinatal period who may have even more limited access to mental health support.

Just over half of the tools (12/22, 54%) included in this review were apps and most were available on both iOS and Android devices. No studies that used a single platform (ie, either iOS or Android, not both) reported their reasoning for doing so, but this likely reflects the high cost of app development and of the smaller feasibility studies that are typically first required to justify and secure funding for large-scale studies, scaling, and wider implementation. Although mHealth has been widely touted as a way to address barriers to care in perinatal populations, restricting access to single platforms may impact equitable delivery of services. Overall, however, increasing smartphone ownership and availability of wireless internet access in public spaces suggest that mHealth tools have the potential to be delivered without restriction to those who have internet access at home.

### Strengths and Limitations

Strengths of this review are its rigorous approach and inclusion of an updated search to capture newly published literature. However, this review did not assess interventions that were available through public app stores and that may not have undergone published scientific evaluation, even though these would also reflect both the current state of mobile tools available for women with perinatal depression or anxiety and the implementation of tools in the public domain. Because only an estimated 6% of the health-based apps have an associated scientific publication, such an endeavor is best suited to its own study [79]. Such a search was beyond the scope of this review, but it could be relevant for future work. This review also excluded tools that were designed to be accessed on stationary devices (ie, computers) but which could, in practice, be accessed on mobile devices. The exclusion of studies in other languages is an additional limitation that biases results to tools likely located in Western, and predominantly English-speaking, contexts.

### Conclusions

Health systems worldwide lack the capacity to fulfill demand for appropriate mental health care services: only 1 in 10 women needing mental health treatment in the perinatal period receive it [80]. eHealth, in general, and mHealth, in particular, have the potential to address the significant barriers to care for women with perinatal depression and/or anxiety and mitigate the negative effects of untreated or undertreated mental illness during this time. This review recommends that future work should incorporate the use of active psychological treatment, address the need for continuity of care across the perinatal period, and include factors that affect long-term clinical sustainability. Results of this review fill an important gap by assisting stakeholders to understand the current state of evidence based mHealth tools for perinatal depression and anxiety. These results can be used to make informed decisions when determining how to develop and implement new or existing tools to fill gaps in knowledge. mHealth tools are part of the future of health care delivery and represent an exciting opportunity to evolve the ways in which psychiatric care is delivered, particularly to women during this vulnerable time.

## Appendix

Multimedia Appendix 1 Overview of studies (n=10) related to mHealth tools for the prevention of perinatal depression and/or anxiety.

Multimedia Appendix 2 Overview of studies related to mHealth tools for the screening (n=6) or treatment (n=6) of perinatal depression and/or anxiety.

## Abbreviations

CBT: cognitive behavioral therapy
eHealth: electronic health
mHealth: mobile health
NICU: neonatal intensive care unit
RCT: randomized controlled trial
