# Supplementary material for: Mobile Health for Perinatal Depression and Anxiety: Scoping Review
Source: J Med Internet Res. 2020 Apr 13;22(4):e17011. doi: 10.2196/17011 (PMC7186872; doi:10.2196/17011)
Supplement: Multimedia Appendix 1 [file jmir_v22i4e17011_app1.docx]

Multimedia Appendix 1. Overview of studies (n=10) related to mHealth tools for the prevention of perinatal depression and/or anxiety

| **1^st^ Author (Year)** | **Population** | **Illness** | **Phase** | **Description of Tool** | **Strategy** | **Mental Health Outcome** |
| --- | --- | --- | --- | --- | --- | --- |
| Jareethum (2008) | Pregnant  N=68 | Anxiety | Feasibility & acceptability | 2 SMS per week with informational support from 28 weeks gestation until birth. | Psychoeducation | Positive difference |
| Song (2013) | Pregnant, Marginalized  N=40 | Depression | Design & development; Feasibility & acceptability | Questions sent via SMS to automated system that can provide an answer or guidance to seek support from a care provider. | Psychoeducation | Positive difference |
| Davis (2014) | Pregnant  N=68 | Depression | Design & development; Feasibility & acceptability | Development of intervention on health behavior changes to deliver 5 SMS per week for three weeks, accompanied by a presentation delivered via iPad. | Psychoeducation | Not reported |
| Dennis-Tiwary (2017) | Pregnant  N=33 | Depression & anxiety | Efficacy & effectiveness | Gamified Attention Bias Modification Training via an app, played over 4 weeks. | Active therapy | No difference |
| Dalton (2018) | Pregnant, Marginalized  N=124 | Depression & anxiety | Feasibility & acceptability | App with information on the 2nd trimester including healthy behaviours, with an emphasis on anxiety and depression. | Psychoeducation | No difference |
| McCarter (2018)  *Protocol* | Postpartum, Marginalized  N=538 | Depression | Feasibility & acceptability; Efficacy & effectiveness | 4 SMS per week on infant care and maternal health with option to request a call from a nurse for 6 months. | Psychoeducation; Communication with healthcare provider | Not reported |
| Biggs (2018) | Unclear | Depression & anxiety | Design & development | Moderated, app-based peer support program. | Peer support | Not reported |
| Chyzzy (2019) | Pregnant & postpartum, Marginalized  N=40 | Depression & anxiety | Feasibility & acceptability | SMS or voice call support from a trained peer from the 3^rd^ trimester to 12 weeks postpartum. | Peer support | Positive difference |
| Fealy (2019)  *Protocol* | Postpartum | Depression & anxiety | Feasibility & acceptability; Efficacy & effectiveness | App with information on newborn and maternal physical and emotional care and a weekly mood rating assessment. | Psychoeducation; Symptom monitoring | Not reported |
| Sun (2019)  *Protocol* | Postpartum | Depression & anxiety | Efficacy & effectiveness | 6 modules on postpartum mental health delivered through CBT principles, completed weekly. | Psychoeducation; Active therapy (CBT) | Not reported |
