# Supplementary material for: Mobile Health for Perinatal Depression and Anxiety: Scoping Review
Source: J Med Internet Res. 2020 Apr 13;22(4):e17011. doi: 10.2196/17011 (PMC7186872; doi:10.2196/17011)
Supplement: Multimedia Appendix 2 [file jmir_v22i4e17011_app2.docx]

Multimedia Appendix 2. Overview of studies related to mHealth tools for the screening (n=6) or treatment (n=6) of perinatal depression and/or anxiety

| **1^st^ Author (Year)** | **Population** | **Illness** | **Phase** | **Description of Tool** | **Strategy** | **Mental Health Outcome** |
| --- | --- | --- | --- | --- | --- | --- |
| **Screening** | | | | | | |
| Jimenez-Serrano (2015) | Postpartum  N=1880 | Depression | Design & development | App to predict depression risk, based on a machine learning algorithm, using questionnaire in the first week postpartum. | Symptom monitoring | Not reported |
| Marcano Belisario (2017) & Doherty (2018)  *Protocol* | Pregnant | Depression | Design & development; Feasibility & acceptability | App for conducting depression assessments, via EPDS plus momentary and contextual questions for 6 months. | Symptom monitoring | Not reported |
| Hantsoo (2017) & Faherty (2018) | Pregnant  N=72 | Depression & anxiety | Feasibility & acceptability; Efficacy & effectiveness | App with psychoeducation, daily and weekly symptom monitoring, and GPS movement tracking. | Psychoeducation; Symptom monitoring; Communication with healthcare provider | Positive difference |
| LaPorte (2017) & Silver (2019) | Pregnant or postpartum  N=210 | Depression & anxiety | Feasibility & acceptability | Computerized screening and SMS thrice weekly for 2 weeks. | Psychoeducation; Symptom monitoring | Not reported |
| Lawson (2019) | Postpartum  N=1000 | Depression | Feasibility & acceptability | Biweekly symptom monitoring plus 3 SMS per week with psychoeducation until 12 weeks postpartum. | Psychoeducation; Symptom monitoring; Communication with healthcare provider | Not reported |
| Ricketts (2019) | Pregnant & postpartum  N=100 | Depression | Feasibility & acceptability | App with symptom monitoring and information on pregnancy and wellness. | Psychoeducation; Symptom monitoring; Communication with healthcare provider | Not reported |
| **Treatment** | | | | | | |
| Broom (2014) & Rhyne (2015) | Postpartum  N=30 | Depression | Feasibility & acceptability | 4 SMS per week on infant care and postpartum depression, some with option to respond and request nurse phone call. | Psychoeducation; Communication with healthcare provider | Not reported |
| Baumel (2018) | Postpartum  N=20 | Depression & anxiety | Feasibility & acceptability; Efficacy & effectiveness | App-based support with trained peers to supplement clinical care plus psychoeducation and mindfulness exercises. | Psychoeducation; Peer support; Active therapy (mindfulness) | Positive difference |
| Teychenne (2018) | Postpartum  N=14 | Depression | Feasibility & acceptability | App with information, exercise motivation, and goal setting support plus treadmill rental for 12 weeks. | Psychoeducation; Other (exercise) | Positive difference |
| Niksalehi (2018) | Postpartum  N=56 | Depression | Efficacy & effectiveness | 2 SMS per day on depression and emotional health for 35 days. | Psychoeducation | Positive difference |
| Green (2019)  *Protocol* | Unclear | Depression | Feasibility & acceptability; Efficacy & effectiveness | Evidence-based CBT intervention delivered by SMS through an AI-enhanced chatbot alongside periodic symptom monitoring. | Communication with healthcare provider; Active therapy (CBT) | Not reported |
| Sawyer (2019) | Postpartum  N=133 | Depression | Efficacy & effectiveness | App with information on infant care, and group chat. | Psychoeducation; Peer support | No difference |
